# The Possible Role of TASK Channels in Rank-Ordered Recruitment of Motoneurons in the Dorsolateral Part of the Trigeminal Motor Nucleus

**DOI:** 10.1523/ENEURO.0138-16.2016

**Published:** 2016-07-20

**Authors:** Keiko Okamoto, Norihito Emura, Hajime Sato, Yuki Fukatsu, Mitsuru Saito, Chie Tanaka, Yukako Morita, Kayo Nishimura, Eriko Kuramoto, Dong Xu Yin, Kazuharu Furutani, Makoto Okazawa, Yoshihisa Kurachi, Takeshi Kaneko, Yoshinobu Maeda, Takashi Yamashiro, Kenji Takada, Hiroki Toyoda, Youngnam Kang

**Affiliations:** 1 Department of Neuroscience and Oral Physiology, Osaka University Graduate School of Dentistry, Suita, Osaka 565-0871, Japan; 2 Department of Orthodontics and Dentofacial Orthopedics, Osaka University Graduate School of Dentistry, Suita, Osaka 565-0871, Japan; 3 Department of Removable Prosthodontics, Osaka University Graduate School of Dentistry, Suita, Osaka 565-0871, Japan; 4 Department of Morphological Brain Science, Graduate School of Medicine, Kyoto University, Kyoto 606-8501, Japan; 5 Department of Pharmacology, Graduate School of Medicine, Osaka University, Suita, Osaka 565-0871, Japan; 6 Department of Vascular Physiology, National Cardiovascular Center Research Institute, Suita, Osaka 565-8565, Japan; 7 Department of Oral Physiology, Graduate School of Medical and Dental Sciences, Kagoshima University, Kagoshima 890-8544, Japan; 8 Department of Oral Anatomy and Cell Biology, Graduate School of Medical and Dental Sciences, Kagoshima University, Kagoshima 890-8544, Japan

**Keywords:** input resistance, leak potassium channel, recruitment, TASK channel, trigeminal motoneuron

## Abstract

Because a rank-ordered recruitment of motor units occurs during isometric contraction of jaw-closing muscles, jaw-closing motoneurons (MNs) may be recruited in a manner dependent on their soma sizes or input resistances (IRs). In the dorsolateral part of the trigeminal motor nucleus (dl-TMN) in rats, MNs abundantly express TWIK (two-pore domain weak inwardly rectifying K channel)-related acid-sensitive-K^+^ channel (TASK)-1 and TASK3 channels, which determine the IR and resting membrane potential. Here we examined how TASK channels are involved in IR-dependent activation/recruitment of MNs in the rat dl-TMN by using multiple methods. The real-time PCR study revealed that single large MNs (>35 μm) expressed TASK1 and TASK3 mRNAs more abundantly compared with single small MNs (15–20 μm). The immunohistochemistry revealed that TASK1 and TASK3 channels were complementarily distributed in somata and dendrites of MNs, respectively. The density of TASK1 channels seemed to increase with a decrease in soma diameter while there were inverse relationships between the soma size of MNs and IR, resting membrane potential, or spike threshold. Dual whole-cell recordings obtained from smaller and larger MNs revealed that the recruitment of MNs depends on their IRs in response to repetitive stimulation of the presumed Ia afferents. 8-Bromoguanosine-cGMP decreased IRs in small MNs, while it hardly changed those in large MNs, and subsequently decreased the difference in spike-onset latency between the smaller and larger MNs, causing a synchronous activation of MNs. These results suggest that TASK channels play critical roles in rank-ordered recruitment of MNs in the dl-TMN.

## Significance Statement

The mastication of foods occurs during the slow-closing phase of the mastication cycle, in which the isometric contraction of jaw-closing muscles is developed through the rank-ordered recruitment of jaw-closing motoneurons (MNs). However, its molecular mechanism remains unknown. Here we show that the TWIK (two-pore domain weak inwardly rectifying K channel)-related acid-sensitive-K^+^ channel (TASK)-1 and TASK3 channels, which determine the input resistance and resting membrane potential, are differentially expressed between small and large MNs, and play critical roles in the rank-ordered recruitment of jaw-closing MNs. The principle of the rank-ordered recruitment was originally proposed based on the conduction velocity of the motor nerve fibers innervating a set of muscle fibers. After a half-century, we for the first time disclosed the molecular mechanism for the rank-ordered recruitment of jaw-closing MNs.

## Introduction

TWIK (two-pore domain weak inwardly rectifying K channel)-related acid-sensitive-K^+^ channel (TASK)-1 and TASK3 channels are the major determinants of the resting membrane potential and input resistance (IR; Lauritzen et al., 2003; Enyedi and Czirják, 2010). TASK1 and TASK3 channel subunits can assemble into dimers; TASK1 homodimeric, TASK3 homodimeric, and TASK1/3 heterodimeric channels (Berg et al., 2004; Enyedi and Czirják, 2010). These channels show different pH sensitivities (Czirják and Enyedi, 2002). The single-channel conductance of TASK3 homodimeric or TASK1/3 heterodimeric channels is two times larger compared with TASK1 homodimeric channels (Kang et al., 2004).

Motoneurons (MNs) in the dorsolateral part of the trigeminal motor nucleus (dl-TMN; Mizuno et al., 1975; Limwongse and DeSantis, 1977) highly express TASK1 and TASK3 mRNAs (Karschin et al., 2001; Talley et al., 2001). During voluntary isometric contraction, a rank-ordered recruitment of motor units occurs, which is well known as the size principle (Henneman et al., 1965; Henneman, 1991). A small motor unit is composed of a small MN and a small number of the thin muscle fibers that it innervates, while a large motor unit is composed of a large MN and a large number of the thick muscle fibers it innervates (Burke, 1975). Such a rank-ordered recruitment of motor units is mediated by the orderly recruitment of MNs, depending on the order of their axonal conduction velocities (Bawa et al., 1984). The axonal conduction velocity of MNs is proportional to their soma sizes, and it is generally believed that the IR in MNs is inversely proportional to their size. Then, it can be assumed that the smaller MNs with larger IRs are more easily activated than the larger MNs with smaller IRs in response to the same synaptic inputs, which is known as the Henneman size principle. However, it was proposed that the specific membrane resistance, but not the cell size itself, is the key determinant of the orderly recruitment of spinal αMNs, although the actual sizes of MNs were not measured (Gustafsson and Pinter, 1984a,b). Despite some controversy about the role of size in the orderly recruitment of MNs, the relationships between the sizes of MNs and IRs, between the sizes of MNs and resting membrane potentials, and between the sizes of MNs and spike thresholds have not been investigated in relation with TASK channels. Because the orderly recruitment of motor units is seen during the voluntary isometric contraction of human jaw-closing (JC) muscles (Yemm, 1977), the MNs located in the dl-TMN (Mizuno et al., 1975; Limwongse and DeSantis, 1977) and innervating JC muscles are likely to be recruited, depending on their sizes or IRs. Then, the expression levels and/or patterns of TASK channels would differ, depending on the sizes of MNs in the dl-TMN.

TASK1 channels are inhibited by various neurotransmitters through the activation of G-protein-coupled receptors (Bayliss et al., 2003; Lesage, 2003). On the other hand, it has been previously reported that TASK1-like leak K^+^ currents in basal forebrain cholinergic neurons are activated by nitric oxide (NO) donor and 8-bromoguanosine-cGMP (8-Br-cGMP; Kang et al., 2007b; Toyoda et al., 2008). It was also found that 8-Br-cGMP enhances TASK1 currents in TASK1-expressed HEK cells, and this effect is brought about by the activation of cGMP-dependent protein kinase (PKG; Toyoda et al., 2010). Nitrergic neurons innervate trigeminal MNs (Abudara et al., 2002), and neuronal NO synthesis is expressed in ∼10% of the premotor neurons projecting to the TMN (Pose et al., 2005). Although the percentage of premotor neurons that express neuronal NO synthesis seems to be low, the numerous nitrergic premotor fibers are in close apposition to trigeminal MN processes (Abudara et al., 2002), and NO is a diffusible gas that readily permeates cell membranes (Bredt et al., 1991). Thus, NO would play an important role in modulating the activity of MNs in the dl-TMN. Provided that the TASK channels are responsible for the rank-ordered recruitment of MNs, it is possible that NO modulates the rank-ordered recruitment of MNs in the dl-TMN.

In the present study, we first investigated whether and how expression levels and/or patterns of TASK channels are different between small and large MNs in the dl-TMN using quantitative real-time PCR and immunohistochemistry. We then examined how MNs in the dl-TMN are recruited by repetitive stimulation of the presumed Ia afferents, and the effects of 8-Br-cGMP on the recruitment of MNs using dual whole-cell current-clamp recordings and voltage-sensitive dye imaging.

## Materials and Methods

All experiments were performed in accordance with the approvals of the animal ethics committees of our institutions for the care and use of laboratory animals.

### Laser capture microdissection and real-time RT-PCR

**Figure 1.**
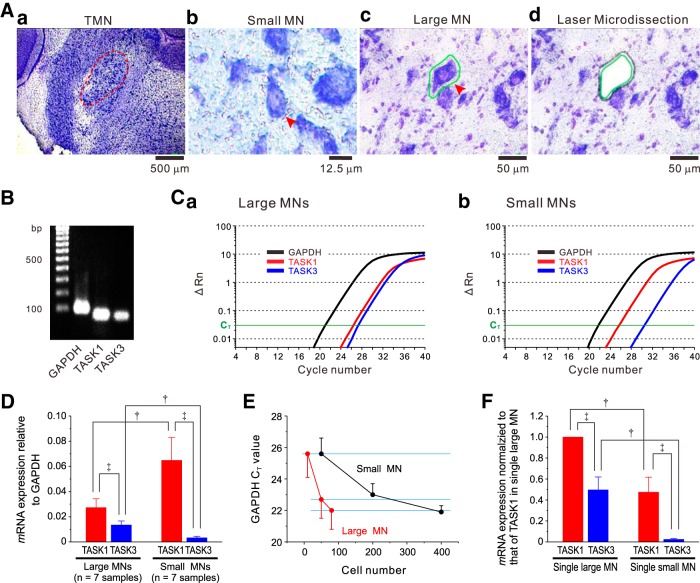
TASK1 and TASK3 mRNA expressions in small and large MNs in the dl-TMN. ***Aa***, Toluidine blue staining of the brainstem slice including the dl-TMN. ***Ab***, ***Ac***, Arrowheads showing small MNs (***Ab***) and large MNs (***Ac***). A margin of the large MN depicted with a green line (***Ac***). ***Ad***, An image of the same section as in ***Ac*** after laser microdissection. ***B***, TASK1 and TASK3 mRNA expression in large MNs revealed by standard RT-PCR. ***Ca***, ***Cb***, Quantitative real-time PCR analysis of TASK1 and TASK3 mRNA expression compared with GAPDH mRNA in large MNs (***Ca***) and small MNs (***Cb***). The ΔRn was plotted against the number of cycles. The mRNA copy number of TASK3 was slightly higher than that of TASK1 in larger MNs, while the former was apparently larger than the latter in small MNs. ***D***, The mean expression levels of TASK1 and TASK3 mRNAs relative to GAPDH mRNA in 80 large MNs and in 400 small MNs (*n* = 7 and *n* = 7 samples, respectively). ‡*p* < 0.001, paired *t* test; †*p* < 0.001, unpaired *t* test. ***E***, The relationships between cell numbers and C_T_ values of GAPDH mRNA obtained from 10, 50, and 80 large MNs (*n* = 7, *n* = 5, and *n* = 5 samples, respectively) and those obtained from 50, 200, and 400 small MNs (*n* = 6, *n* = 6, and *n* = 5 samples, respectively). ***F***, The expression levels of TASK1 and TASK3 mRNAs in a single large MN and those in a single small MN. The respective mRNA expression levels were normalized to that of TASK1 mRNA in a single large MN. ‡*p* < 0.001, paired *t* test; †*p* < 0.001, unpaired *t* test.

Wistar rats of both sexes at postnatal days 15–17 (Nihon Dobutsu) were anesthetized with chloral hydrate (400 mg/kg, i.p.) and transcardially perfused with PBS. The brain was quickly removed from the skull and immediately flash frozen with dry ice. Serial cryostat sections (20 µm) of the dl-TMN were cut and mounted on polyethylene terephthalate (PET) membrane slides (Leica Microsystems). Sections were air dried at room temperature for 30 s, fixed with 70% ethanol for 1 min, washed in RNase-free diethylpyrocarbonate (DEPC)-treated water and stained with 0.05% toluidine blue for 1 min (Fig. 1*Aa*). After rinsing twice in DEPC water for 1 min, the sections were air dried for 5 min and immediately subjected to laser microdissection (LMD).

PET slides were mounted on a Leica AS LMD System (Leica Microsystems) with the section facing downward. Dye-labeled MNs were selected for capture and divided into two groups according to their mean diameters. The neuron whose mean diameter was 15–20 µm was categorized as a small MN (Fig. 1*Ab*), while the neuron whose mean diameter was >35 µm was categorized as a large MN (Fig. 1*Ac*). After adjusting intensity, aperture, and cutting velocity, the pulsed UV laser beam was carefully directed along the border of the neuron (Fig. 1*Ad*). The cut area was then transferred by gravity alone into a microcentrifuge tube cap placed directly underneath the section. The tube cap was filled with a guanidine isothiocyanate-containing buffer (Buffer RLT, Qiagen) to ensure the isolation of intact RNA. For each experiment, 80 large MNs were sampled from one to two brains, and 400 small MNs were sampled from two to three brains. The 80 large or 400 small MNs were collected in one microcentrifuge tube as one sample (see below).

Total RNA was extracted using the RNeasy Micro Kit (Qiagen) according to the manufacturer instructions, during which samples were subjected to DNase digestion using DNase I (Qiagen) to remove any contaminating genomic DNA. RNA was then reverse transcribed for 1 h at 37°C using Sensicript Reverse Transcription kit (Qiagen). Real-time PCR was performed in a reaction mixture (20 µl) composed of 10 µl of 2× Fast SYBR Green Master Mix (Applied Biosystems), 0.35 mm each primer and 2 µl of reverse transcription product, through the following series of reactions: 95°C for 20 s, followed by 40 cycles (95°C, 3 s; 60°C, 30 s), with a Step One Real-Time PCR System (Applied Biosystems). The following primers were used for PCR: rat TASK-1: forward, 5'-CGGCTTCCGCAACGTCTAT-3', and reverse, 5'-TTGTACCAGAGGCACGAGCA-3'; rat TASK-3: forward, 5'-GACGTGCTGAGGAACACCTACTT-3', and reverse, 5'-GTGTGCATTCCAGGAGGGA-3'; and GAPDH: forward, 5'-GAGAATGGGAAGCTGGTCATCAAC-3' and reverse, 5'-ACTCCACGACATACTCAGCACCAG-3'. Standard curves were generated for each set of primers using serial dilutions of rat brain cDNA to ensure a similar efficiency of amplification (TASK1, 100%; TASK3, 100%). All the primers were obtained from Sigma Genosys. Aliquots of the PCR products were analyzed by 2% agarose gel electrophoresis (Nacalai Tesque) and were visualized using ethidium bromide. The PCR product was run in parallel with known molecular weight markers (100 bp ladder, Bio-Rad). The amplified PCR fragments obtained from large MNs were detected in accordance with the respective lengths predicted by the primers (Fig. 1*B*). The data were analyzed using the comparative cycle-threshold (C_T_) method (Pfaffl, 2001), where the amount of target is normalized to an endogenous reference gene, GAPDH.

### Evaluation of size-dependent expression of GAPDH mRNA

Because the housekeeping gene GAPDH mRNA level is likely to be proportional to the cell volume, the GAPDH mRNA levels obtained from a certain number of small MNs (N_S_) and a certain number of large MNs (N_L_) are proportional to N_S_ × V_S_ and N_L_ × V_L_, respectively, given that V_S_ and V_L_ are the mean cell volumes of the sampled small and large MNs, respectively. When N_S_ × V_S_ is equal to N_L_ × V_L_, V_S_/V_L_ is equal to N_L_/N_S_. The normalized GAPDH mRNA in a single small MN compared with that in a single large MN is proportional to N_L_/N_S_. Therefore, we evaluated the relationship between the cell number and C_T_ of GAPDH mRNA obtained from 50, 200, and 400 small MNs (*n* = 6, *n* = 6, and *n* = 5 samples, respectively) and that between the cell number and C_T_ of GAPDH mRNA obtained from 10, 50, and 80 large MNs (*n* = 7, *n* = 5, and *n* = 5 samples, respectively). C_T_ represents the fractional cycle number at which the baseline-corrected normalized fluorescence signal (*Δ*Rn) reaches a threshold value (0.03).

### Anti-TASK1 antibody

Peptide YKSREKLQYSIPMIIPRDLSTSDTC, which corresponds to C-terminal 25 aa (residues 332–356) of rat TASK1 (Leonoudakis et al., 1998) was synthesized (ThermoFisher Scientific). Cysteine in the C-terminal of the peptide was used for conjugation by a maleimide method. The peptide was conjugated with an equal weight of maleimide-activated bovine serum album (ThermoFisher Scientific). Four female guinea pigs (200 g; Shimizu Experimental Materials) were immunized by intracutaneous injections of the conjugate (0.5 mg/animal) in Freund’s complete adjuvant (Difco), and, 4 weeks later, in incomplete adjuvant. The sera were recovered 14 days after the second immunization. The antibody was purified by ammonium sulfate fractionation (50% saturation), followed by affinity chromatography on a SulfoLink gel (ThermoFisher Scientific) coupled with peptide (2 mg peptide/ml gel). The antibody was eluted with 0.1 m glycine-HCl, pH 2.5, and 0.1 m tetraethyl ammonium, pH 11.8, and was used for the following experiments.

The specificity of anti-TASK1 antibody was examined using HEK293 cells transfected with TASK1 or mock. For the transient expression of TASK1, HEK293 cells (DS Pharma Biomedical) cultured in DMEM supplemented with 10% FBS, 100 U/ml penicillin, and 100 µg/ml streptomycin were transfected with pIRES2-ZsGreen1-TASK1 by using Lipofectamine LTX (Invitrogen) or the empty vector pIRES2-ZsGreen1 (mock; Toyoda et al., 2010). For immunostaining of transient transfected cell lines, cells plated onto glass coverslips 4 h after transfection were fixed with 3% formaldehyde, 75% saturated picric acid, and 0.1 m phosphate-NaOH buffer at pH 7.2. The cells were stored at 4°C in PBS containing 0.02% sodium azide. The cells were incubated overnight with 1 μg/ml affinity-purified guinea pig anti-rat TASK1 antibody in PBS containing 0.3% Triton X-100, 0.12% λ-carrageenan, 0.02% sodium azide, and 1% normal donkey serum (PBS-XCD); and then were incubated for 1 h with 10 μg/ml biotinylated donkey anti-guinea pig IgG antibody (RRID: AB[lowen]2341097, Jackson ImmunoResearch) in PBS-XCD. Subsequently, the cells were incubated for 1 h with avidin–biotin enzyme complex (ABC; VECTASTAIN Elite ABC, Vector Laboratories) in PBS containing 0.3% Triton X-100 (PBS-X). Furthermore, the sections were incubated for 30 min with biotinylated tyramine (BT)-glucose oxidase (GO) reaction mixture containing 1.25 μm BT, 3 μg/ml GO (259 U/mg; Nacalai Tesque), 2 mg/ml β-d-glucose, and 1% BSA in 0.1 m PB, pH 7.4. After a rinse with PBS-X, the sections were incubated for 1 h with 5 μg/ml Alexa Fluor 594-conjugated streptavidin (RRID: AB[lowen]2313574; Invitrogen) to stain TASK1 and then were incubated for 5 min with DAPI to stain nuclei.

### Fluorescence immunohistochemistry

**Figure 2.**
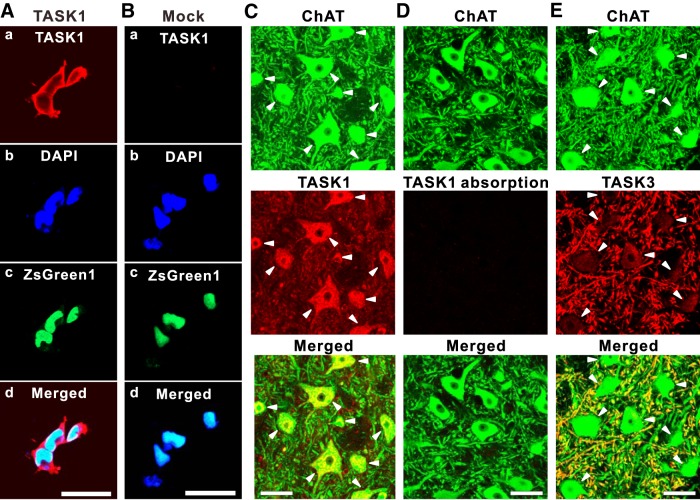
Complementary distribution of TASK1 and TASK3 channels in MNs in the dl-TMN. ***Aa–Ad***, HEK cells transfected with TASK1 and ZsGreen1 displaying immunoreactivity along the plasma membrane and partly in the cytoplasm (***Aa***); nuclei staining with DAPI (***Ab***); TASK1 transfection ensured with ZsGreen1 (***Ac***); and a merged fluorescence image (***Ad***). ***Ba–Bd***, HEK cells transfected with mock and ZsGreen1 displaying no immunoreactivity (***Ba***); nuclei staining with DAPI (***Bb***); mock transfection ensured with ZsGreen1 (***Bc***); and a merged fluorescence image (***Bd***). ***C***, Confocal photomicrographs showing immunoreactivity for ChAT (green) and TASK1 (red) in MNs. As revealed in the merged image, the TASK1 immunoreactivity was seen in somata (filled arrowheads) but not in dendrites. ***D***, Confocal photomicrographs showing the elimination of TASK1 immunoreactivity in ChAT (green)-positive MNs following the absorption of anti-TASK1 antibody by preincubating with the antigen peptide of TASK1. ***E***, Confocal photomicrographs showing immunoreactivity for ChAT (green) and TASK3 (red) in MNs. As revealed in the merged image, the TASK3 immunoreactivity was primarily seen in dendrites but not in somata (filled arrowheads). Scale bars: ***A*–*E***, 50 μm.

For immunostaining, fixed brains were prepared from three Wistar rats following the protocol described in the previous study (Toyoda et al., 2010). The 40-μm-thick coronal sections of the fixed brains were incubated overnight in PBS-XCD with 2 μg/ml anti-rat TASK1 guinea pig antibody or 1 μg/ml anti-rat TASK3 (residues 57–73) rabbit antibody (RRID: AB[lowen]2039953, Alomone Labs) with or without anti-ChAT goat antiserum [AB144P (RRID:AB[lowen]2079751), Chemicon] diluted 1:500. After a wash with PBS, the sections were incubated for 2 h with 10 μg/ml biotinylated anti-guinea pig IgG donkey antibody (Jackson ImmunoResearch) or 10 μg/ml biotinylated anti-rabbit IgG donkey antibody (RRID: AB[lowen]2340593; Jackson ImmunoResearch) and then incubated for 1 h with ABC in PBS-X. For the immunostaining of TASK1, the sections were then incubated for 30 min with BT-GO reaction mixture, followed by incubation for 1 h with 5 μg/m Alexa Fluor 594-conjugated streptavidin. For the immunostaining of TASK3, the sections were then incubated for 30 min with TSA Cyanine 3 (Cy3) System (PerkinElmer). For the immunostainings of ChAT, the sections were then incubated for 2 h with 10 μg/ml Alexa Fluor 488-conjugated anti-goat IgG donkey antibody (RRID: AB[lowen]2534102; Invitrogen) and Cy3-conjugated streptavidin (Invitrogen) in the presence of 10% normal rabbit serum. For control experiments, when one of the primary antibodies was omitted or replaced with normal IgG or serum, no immunofluorescence for the omitted or replaced antibody was detected. For the absorption tests for anti-TASK1 and anti-TASK3 antibodies, the primary antibody solution was preincubated for 1 h with the antigen peptide (1 μg peptide for 1 μg antibody) prior to the incubation with sections (Fig. 2*D*, TASK1 absorption test). The sections were observed with a confocal laser-scanning microscope (LSM510, Zeiss). Alexa Fluor 488 and 594 were excited with 488 and 543 nm laser beams, and observed through emission filters of 505–530 and >560 nm, respectively. The digital images were captured using LSM510 software (Zeiss).

### Slice preparation

The procedure for slice preparation was the same as that in the previous study (Kang et al., 2007a). Using Wistar rats of both sexes at postnatal days 7–14 (Nihon Dobutsu), coronal sections with 250 μm thickness, including the dl-TMN, were cut.

### Whole-cell recording from MNs in the dl-TMN

**Figure 3.**
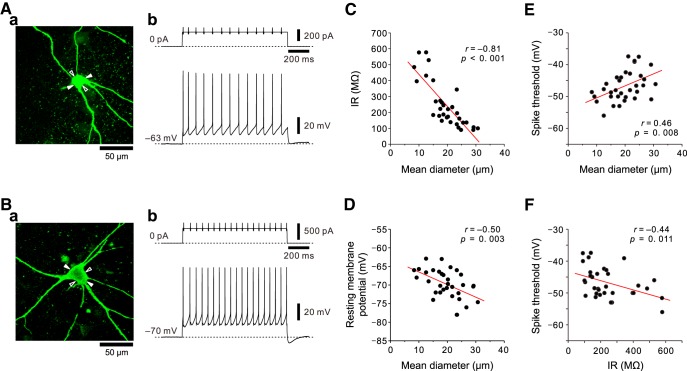
Relationships between the sizes of MNs in the dl-TMN and IRs, resting membrane potentials, or spike thresholds. ***Aa***, ***Ba***, Small (19 × 17 μm) and large (30 × 21 μm) MNs filled with biocytin (***Aa*** and ***Ba***, respectively). The minor and major axes are indicated with open and filled arrowheads, respectively. ***Ab***, ***Bb***, Membrane potential responses to depolarizing current pulses applied to small and large MNs at the resting membrane potential (***Ab*** and ***Bb***, respectively). ***C***, The inverse relationship between the sizes of MNs and IRs (*n* = 33). ***D***, The inverse relationship between the sizes of MNs and resting membrane potentials (*n* = 33). ***E***, The positive relationship between the sizes of MNs and spike thresholds (*n* = 33). ***F***, The inverse relationship between the IRs and spike thresholds (*n* = 33).

**Figure 4.**
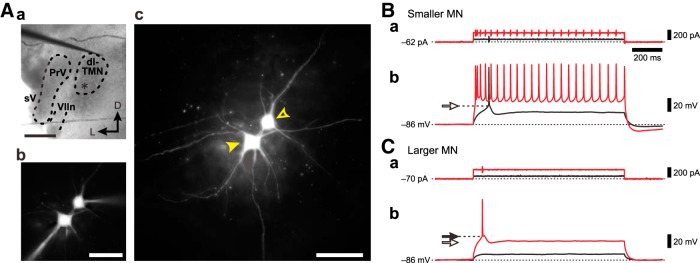
Membrane potential responses to depolarizing current pulses in smaller and larger MNs in the dl-TMN. ***Aa***, A brainstem slice including the dl-TMN. A stimulation electrode was placed just dorsal to the TMN. PrV, Trigeminal principle nucleus; VIIn, facial nerve; sV, sensory root of the trigeminal nerve. Scale bar, 500 μm. ***Ab***, A simultaneous recording obtained from a pair of smaller and larger MNs that were filled with Lucifer yellow. Scale bar, 100 μm. ***Ac***, A Lucifer yellow image of the smaller and larger MNs after fixation with paraformaldehyde (open and filled arrowheads, respectively). Scale bar, 100 μm. ***B***, ***C***, Membrane potential responses to depolarizing current pulses applied to the smaller and larger MNs (***Bb*** and ***Cb***, respectively) from the same baseline membrane potential of −86 mV brought about by negative DC current injection of −62 and −70 pA, respectively (***Ba*** and ***Ca***, respectively). The apparent spike threshold was lower in the smaller MN (open arrow) than in the larger MN (filled arrow).

**Figure 5.**
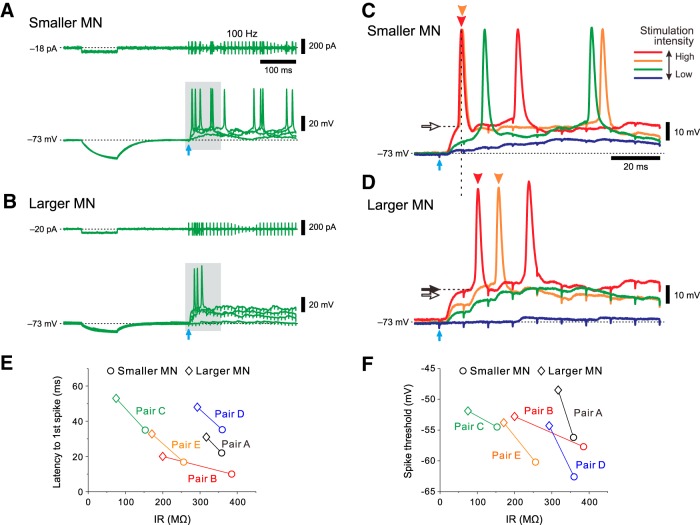
IR-dependent activation/recruitment of MNs in the dl-TMN. ***A***, ***B***, Membrane potential responses to hyperpolarizing current pulses and those evoked by 100 Hz stimulation of presumed Ia afferents in the smaller and larger MNs (***A*** and ***B***, respectively). ***C***, ***D***, The enlarged traces of the responses (gray area in ***A*** and ***B***, respectively) in the smaller and larger MNs (***C*** and ***D***, respectively). ***E***, The relationship between IRs and latencies to the first spikes in five pairs of MNs of different sizes when the difference in the latency to the first spike between the pair of smaller and larger MNs became maximum. ***F***, The relationship between IRs and spike thresholds in five pairs of MNs of different sizes.

**Figure 6.**
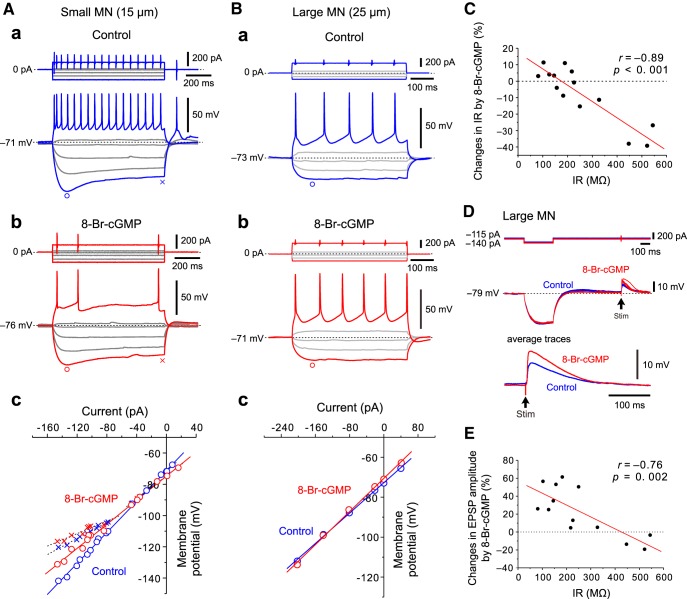
Modulation of IRs by 8-Br-cGMP in small and large MNs in the dl-TMN. ***Aa***, ***Ab***, Sample traces of responses to depolarizing and hyperpolarizing current pulses obtained before application (***Aa***) and during application (***Ab***) of 8-Br-cGMP in a small MN. ***Ac***, The *I–V* relationships obtained before and during application of 8-Br-cGMP in a small MN. The *I–V* relationships measured at the peak sag potentials are almost linear (blue and red circles), while those measured just before the offset of current pulses are inwardly rectifying (blue and red crosses). ***Ba***, ***Bb***, Sample traces of responses to depolarizing and hyperpolarizing current pulses obtained before application (***Ba***) and during application (***Bb***) of 8-Br-cGMP in a large MN. ***Bc***. The *I–V* relationships obtained before and during the application of 8-Br-cGMP in a large MN. ***C***, The relationship between IRs of MNs and changes in IRs by 8-Br-cGMP (*n* = 14). ***D***, EPSPs evoked by stimulation just dorsal to the TMN obtained before and during the application of 8-Br-cGMP in a large MN. ***E***, The relationship between IRs and changes in EPSP amplitudes by 8-Br-cGMP (*n* = 13).

**Figure 7.**
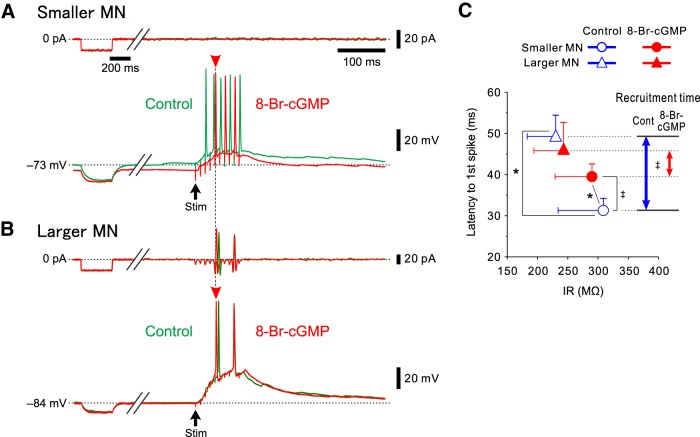
8-Br-cGMP differentially modulates spike-onset latencies between smaller and larger MNs in the dl-TMN. ***A***, ***B***, Membrane potential responses to hyperpolarizing current injections and those evoked by 100 Hz stimulation of presumed Ia afferents in smaller and larger MNs (***A*** and ***B***, respectively) obtained before (green traces) and during the application of 8-Br-cGMP (red traces). ***C***, The relationship between the IRs and the latencies to first spikes obtained before and after the application of 8-Br-cGMP in four pairs of MNs of different sizes. The blue and red double-headed arrows represent the recruitment time, calculated as the difference in the mean latency to the first spike between the smaller and larger MNs obtained before and after the application of 8-Br-cGMP, respectively. *p < 0.05, Wilks’ lambda; ‡*p* < 0.05, paired *t* test.

The electrophysiological studies were performed on the MNs in the dl-TMN. Axopatch 200B (MDS Analytical Technologies; Figs. 3–6) and Axoclamp 2B (MDS; Fig. 7) were used for whole-cell patch-clamp recordings. The standard extracellular solution had the following composition (in mm): 124 NaCl, 1.8 KCl, 2.5 CaCl_2_, 1.3 MgCl_2_, 26 NaHCO_3_, 1.2 KH_2_PO_4_, and 10 glucose, bubbled with a mixture of 95% O_2_/5% CO_2_. The internal solution had the following composition (in mm): 123 K-gluconate, 8 KCl, 20 NaCl, 0.5 MgCl_2_, 2 ATP-Na_2_, 0.3 GTP-Na_3_, 10 HEPES, and 0.1 EGTA; the pH was adjusted to 7.3 with KOH. In some experiments, 123 mm K-gluconate, 8 mm KCl, and 20 mm NaCl were substituted for 118 mm K-gluconate, 18 mm KCl, 14 mm NaCl, and 10 mm biocytin to characterize the morphology of MNs. The patch pipettes had a DC resistance of 4–5 MΩ when filled with the internal solution. The membrane potential values given in the text were corrected for the junction potential between the internal solution (negative) and the standard extracellular solution (10 mV). All recordings were made at room temperature (20–24°C). The sealing resistance was usually >10 GΩ. Whole-cell currents or voltages were low-pass filtered at 2 kHz (four-pole Bessel filter) and digitized at a sampling rate of 10 kHz (Digidata 1322A, MDS Analytical Technologies). Under the current-clamp condition, depolarizing and hyperpolarizing current pulses were applied every 30 s. The IR was calculated from the *I–V* relationships, which were usually linear when measured at the peak timing of the hyperpolarizing responses or sag potentials evoked immediately after the onset of negative current pulses (Fig. 6*Aa*, blue circle). Microstimulation of 100 μs duration was delivered via a sharp monopolar tungsten electrode (DC resistance, 1 MΩ), which was placed just dorsal to or in the dorsal part of the TMN to simulate the presumed Ia input arising from the mesencephalic trigeminal sensory neurons (Fig. 4*Aa*). The united or stem axons of the mesencephalic trigeminal sensory neurons, which bifurcate into the peripheral axons innervating muscle spindles and the central axons projecting into the TMN, are located just dorsal to the TMN (Shigenaga et al., 1988, 1990). The intensity of the stimulus current applied to the dorsal part of the TMN was <5 μA. The direct current spread is very confined within a small area as the amplitude of the maximum response evoked at a site around the tip of the stimulating electrode immediately after stimulation sharply decreases by half at a distance of 80 μm and to almost zero at <200 μm from the tip of the stimulating electrode in slices (Sato et al., 2008). Thus, the stimulus current does not spread over the entire dl-TMN (500 × 1000 μm). 8-Br-cGMP (a membrane permeable analog of cGMP, which is the second messenger of NO; Sigma-Aldrich) was bath applied at a concentration of 100 μm.

### Preparation of *Xenopus* oocytes expressing TASK1 and TASK3 channels

The isolation and maintenance of the oocytes of frogs (*Xenopus laevis*) and injection with cRNA were performed as described previously (Inanobe et al., 2001). Mouse TASK1 and TASK3 cDNAs were obtained from pIRES2-ZsGreen1-TASK1 (Toyoda et al., 2010) and pIRES2-ZsGreen1-TASK3, respectively. Mouse TASK1 and TASK3 cDNAs were subcloned into pcDNA3.1(–) vector under the control of the T7 promotor and were subjected to cRNA preparation. Straight-chain DNAs that were obtained by truncating the downstream sequences of circular plasmids of TASK1 and TASK3 were used as templates. The cRNAs for injection into oocytes were prepared with T7 RNA polymerase (Invitrogen). Oocytes were injected with cRNAs for TASK1 (50 ng/oocyte) and TASK3 (50 ng/oocyte). After injection, oocytes were incubated at 18°C in ND96 solution, which contained the following (in mm): 96 NaCl, 2 KCl, 1.8 CaCl2, 1 MgCl_2_, and 5 HEPES, pH 7.4 with NaOH, and supplemented with gentamicin (50 μg/ml). Currents were recorded 1–3 d after the cRNA injection.

### Two-electrode voltage-clamp recordings from *Xenopus* oocytes

Membrane currents of oocytes were recorded by two-electrode voltage clamp using the GeneClamp 500 amplifier (MDS) at room temperature. Data were reproduced and analyzed with pCLAMP version 10 (MDS) and Clampfit version 10.2 (MDS). The bath solution contained the following (in mm): 90 KCl, 3 MgCl_2_, 0.15 niflumic acid, and 5 HEPES, pH 7.4 with KOH. The tip resistance of the glass electrodes was 0.4–1.5 MΩ when filled with the 3 m KCl pipette solution. Niflumic acid (0.1 mm; Sigma-Aldrich) was added in the bath solution to block Ca^2+^-dependent Cl^−^ channels, which are richly expressed in the plasma membrane of oocytes (Dascal, 1987). 8-Br-cGMP was bath applied at concentration of 100 μm. The currents were measured 5–10 ms before the offset of the voltage step.

### Optical recording using RH414

The procedures for the optical recording and its data analysis were previously described (Sato et al., 2008). Briefly, the slices were stained with 200 μm N-(3-triethylammoniumpropyl)-4-(4-(4- (diethylamino) phenyl) butadienyl) pyridinium dibromide (RH414, Molecular Probes; Grinvald et al., 1984). The stained slices were illuminated by light with a wavelength of 535 ± 15 nm using a stabilized 150 W xenon lamp. The fluorescence emitted from RH414 was long-pass filtered above 580 nm and measured with a CCD camera (NeuroCCD-sm, RedShirtImaging), which was attached to a microscope (BX-51WI, Olympus) equipped with a water-immersion objective (10×, 0.3 numerical aperture; Olympus). The imaged area was 1.6 × 1.6 mm^2^, and each pixel (element) of the 80 × 80 array detected the optical signals generated by a square region (20 × 20 μm^2^). The fluorescence images were captured at a sampling rate of 1000 Hz. Repetitive electrical stimulation (10 pulses at 100 Hz) of 100 μs duration was delivered via a sharp monopolar tungsten electrode (DC resistance, 1 MΩ) placed just dorsal to or in the dorsal part of the TMN to simulate presumed Ia afferents (Fig. 9*A*). 8-Br-cGMP was bath applied at a concentration of 100 μm. The amplitudes of the 1st to 9th (before 8-Br-cGMP application) or the 10th (after 8-Br-cGMP application) optical responses at the region of interest placed in the dl-TMN (Fig. 9*A*) were normalized to that in the 10th response obtained before the application of 8-Br-cGMP (Fig. 9*D*).

### Data analysis

Statistical analysis was performed using STATISTICA10J (StatSoft). Numerical data were expressed as the mean ± SD. The statistical significance was assessed using ANOVA followed by Fisher’s PLSD *post hoc* test, paired and unpaired Student’s *t* test, Pearson’s correlation coefficients, Wilcoxon signed rank test, and Wilks’ lambda. The Student’s *t* test was used when the data showed a normal distribution, as confirmed by the Kolmogorov–Smirnov test. Statistical results are given as a *p* value, except when it is very small (*p* < 0.001). A *p* value <0.05 was considered statistically significant.

## Results

### The expression levels of TASK1 and TASK3 mRNAs in small and large MNs in the dl-TMN

First, we quantified the expression levels of TASK1 and TASK3 mRNAs in small (15–20 μm) and large (>35 μm) MNs in the dl-TMN using a real-time PCR method. The representative example of quantitative PCR for TASK1, TASK3, and GAPDH mRNAs obtained from 80 large MNs and that obtained from 400 small MNs are shown in Figure 1, *Ca* and *Cb*. Under the condition that the PCR amplification efficacy was the same between the TASK1 and TASK3 primers (see Materials and Methods), the expression level of TASK1 mRNA was estimated to be two times higher than that of TASK3 mRNA in large MNs (*p* < 0.001, paired *t* test; *n* = 7 samples), while the former was 20 times higher than the latter in small MNs (*p* < 0.001, paired *t* test; *n* = 7 samples; Fig. 1*D*).

As revealed by the relationship between the cell number and the C_T_ of GAPDH mRNA (Fig. 1*E*), the mean C_T_ value obtained from 50 or 400 small MNs was almost the same or very slightly smaller than that obtained from 10 or 80 large MNs, respectively, while that obtained from 200 small MNs was slightly larger than that obtained from 50 large MNs. This suggests that the GAPDH content in a single large MN is at least more than four times and very close to five times larger than that in a single small MN. This value is consistent with the volume ratio between the mean cell volumes of the small and large MNs: compared to the volume ratio (>5.4 times) of large spheres to small ones (diameter ratio >35/20), the volume ratio of large polygonal neurons to small ones can be smaller, depending on their shapes (35/20 > axis ratio > [35 × cos (θ_1_)]/[20 × cos (θ_2_)], when θ_1_ is larger than θ_2_; 0 < θ < 90˚), given that polygonal shape is a quadrangular pyramid. Based on the assumption that GAPDH content in a single large MN is five times larger than that in a single small MN, we estimated the expression levels of TASK1 and TASK3 mRNAs in single large and small MNs. The expression level of TASK1 mRNA in one large MN was estimated to be 2.1 times higher than that in one small MN (*p* < 0.001, unpaired *t* test; *n* = 7 samples), while that of TASK3 mRNA in one large MN was estimated to be 20 times higher than that in one small MN (*p* < 0.001, unpaired *t* test; *n* = 7 samples; Fig. 1*F*), suggesting that the IR in one large MN is much smaller than that in one small MN.

### Subcellular distributions of TASK1 and TASK3 channels in MNs in the dl-TMN

As we made a new antibody to TASK1 in the present study (see Materials and Methods), we first examined its selectivity using HEK293 cells transfected with TASK1 or mock together with ZsGreen1. In the HEK293 cells represented by DAPI staining (Fig. 2*Ab*,*Bb*), TASK1 or mock transfection was ensured by ZsGreen1 expression (Fig. 2*Ac*,*Bc*). In the HEK293 cells transfected with TASK1, the strong TASK1 immunoreactivity was seen along the plasma membrane and partly in the cytoplasm (Fig. 2*Aa*), while the HEK293 cells transfected with mock displayed no TASK1 immunoreactivity (Fig. 2*Ba*). These results suggest that this antibody is specific to the TASK1 protein heterologously expressed in HEK cells.

We then examined TASK1 immunoreactivity in MNs in the dl-TMN. In ChAT-positive MNs, their somata were strongly immunoreactive to anti-TASK1 antibody (Fig. 2*C*, filled arrowheads), whereas their neuropils were very weakly immunoreactive to the antibody (Fig. 2*C*). When the anti-TASK1 antibody was absorbed by preincubating with the antigen peptide of TASK1, the TASK1 immunoreactivity was seen neither in TMN (Fig. 2*D*) nor in the other brain regions where TASK1 immunoreactivity was seen, as follows. The distribution pattern of TASK1 immunoreactivity was found to be remarkably similar to that demonstrated using *in situ* hybridization in previous studies (Karschin et al., 2001; Talley et al., 2001). Strong TASK1 immunoreactivity was found not only in the trigeminal MNs but also in hypoglossal and facial MNs. The moderate TASK1 immunoreactivity to this antibody was found in neurons in barrel and insular cortices, cerebellar granule layers, the nucleus of solitary tract, and intermediate reticular nucleus, while the spinal tract nucleus of trigeminal nerve and superior cerebellar peduncle displayed weak immunoreactivity. Thus, there were three lines of evidence that ensured the specificity of the TASK1 antibody. In contrast, somata of ChAT-positive MNs were almost immunonegative to anti-TASK3 antibody (Fig. 2*E*, filled arrowheads), while their neuropils showed intense TASK3 immunoreactivity (Fig. 2*E*). Thus, TASK1 immunoreactivity was seen almost exclusively in the somata, while TASK3 immunoreactivity was seen only in dendrites instead of the somata, as shown in the middle panels of Figure 2, *C* and *E*. The merged images clearly show that the somata, instead of the dendrites, are shown as yellow (i.e., green plus red) in the bottom panel of Figure 2*C*, while the dendrites, instead of the somata, are shown as yellow (i.e., green plus red) in the bottom panel of Figure 2*E*. These results clearly indicate that TASK1 and TASK3 are complementarily expressed in somata and dendrites, respectively, regardless of the size of the MNs in the dl-TMN.

### Inverse relationship between the size of MNs in the dl-TMN and IRs

We next investigated the relationships between the sizes of αMNs in the dl-TMN and IRs, resting membrane potentials, or spike thresholds. Whole-cell current-clamp recordings were obtained from 33 MNs that displayed polygonal and stellate-shaped somata with many primary dendrites when observed under Nomarski optics (63×; Fig. 3). The membrane potential responses were evoked by the injection of depolarizing and hyperpolarizing current pulses at resting membrane potentials (Fig. 3*Ab*,*Bb*). After the recordings, MNs filled with biocytin were fixed with 4% paraformaldehyde, and the sizes of the labeled neurons were evaluated with a confocal microscope image (Fig. 3*Aa*,*Ba*). The morphological features of these multipolar αMNs were distinct from those of γMNs, which show piriform or round-shaped somata with fewer primary dendrites (Card et al., 1986; Bae et al., 2002).

The IRs and resting membrane potentials were inversely correlated with the sizes of MNs, as revealed by their significant negative correlations (Fig. 3*C*,*D*, respectively), while the spike threshold was significantly positively correlated with the sizes of MNs (Fig. 3*E*). Subsequently, the spike threshold was significantly inversely correlated with the sizes of MNs (Fig. 3*F*). These results indicate that smaller MNs in the dl-TMN have larger IRs, depolarized resting membrane potentials, and lower spike thresholds, while larger MNs in the dl-TMN have smaller IRs, hyperpolarized resting membrane potentials, and higher spike thresholds. In the next series of experiments, we examined whether MNs in the dl-TMN are recruited based on their soma sizes or IRs using simultaneous whole-cell current-clamp recordings from a pair of MNs of different size.

### The orderly activation of MNs in the dl-TMN

In the dl-TMN, simultaneous whole-cell recordings were obtained from a pair of randomly selected MNs that are located close each other to avoid the differences in the numbers of synaptic inputs and postsynaptic receptors in the slice preparations, one of which is relatively larger or smaller than the other (Fig. 4*Ab*,*Ac*, open and filled arrows, respectively). In such a pair of MNs, membrane potential responses to the depolarizing current pulses were examined at the same baseline membrane potential of −86 mV brought about by negative DC current injections of −62 and −70 pA, respectively (Fig. 4*Ba*,*Ca*, respectively). When a depolarizing current pulse with the same small intensity was applied to the pair of smaller and larger MNs (Fig. 4*Ba*,*Ca*, black traces), a spike was evoked only in the smaller MN (Fig. 4*Bb*, black trace) but not in the larger MN (Fig. 4*Cb*, black trace). When a depolarizing current pulse with the same large intensity was applied to the pair of the smaller and larger MNs (Fig. 4*Ba*,*Ca*, red traces), a repetitive spike firing was evoked in the smaller MN (Fig. 4*Bb*, red trace), while only a single spike was evoked in the larger MN (Fig. 4*Cb*, red trace). The spike threshold in the smaller MN was apparently lower than that in the larger MN (Fig. 4*Bb*,*Cb*, compare open arrows, filled arrows).

We further compared their responses to hyperpolarizing current pulses and repetitive electrical stimulation at 100 Hz, which was applied to the area just dorsal to the TMN, between the smaller and larger MNs. The responses to hyperpolarizing current pulses were apparently larger in the smaller MN than in the larger MN, suggesting that the IR in the smaller MN was larger than that in the larger MN (Fig. 5*A*,*B*). As the stimulus intensity was increased, EPSPs evoked by 100 Hz stimulation increased in amplitude in both the smaller and larger MNs, and were temporally summated to consequently trigger spikes. However, the first spiking invariably occurred at an earlier time in the smaller MN than in the larger MN, regardless of stimulus intensity (Fig. 5*C*,*D*, red, orange arrowheads). The spike threshold in the smaller MN was apparently lower than that in the larger MN (Fig. 5*C*,*D*, compare open arrows, filled arrows), consistent with the observation made in response to the depolarizing current pulses (Fig. 4*B*,*C*).

The pooled data analysis of the simultaneous whole-cell recordings obtained from five pairs of MNs of different sizes revealed that the latency to the first spike was invariably shorter in a smaller MN than in a larger MN (Fig. 5*E*) and that the spike threshold was invariably lower in a smaller MN than in a larger MN (Fig. 5*F*). The median of the latency to the first spike obtained from smaller MNs (22 ms) was significantly shorter (*p* = 0.043, Wilcoxon signed rank test) than that obtained from larger MNs (32.8 ms; Fig. 5*E*). The median of the spike threshold obtained from smaller MNs (−57.7 mV) was significantly lower (*p* = 0.043, Wilcoxon signed rank test) than that obtained from larger MNs (−52.8 mV; Fig. 5*F*). Given the pair recordings from a neuronal population in which the inverse relationships were observed between IRs and latencies to the first spikes or spike thresholds, these results suggest that MNs in the dl-TMN are recruited in an orderly manner, depending on their sizes or IRs.

It has been shown previously that TASK1-mediated leak K^+^ currents in basal forebrain cholinergic neurons were activated by NO signaling through the cGMP/PKG transduction pathway (Toyoda et al., 2010). Cholinergic neurons located in the pedunculopontine and laterodorsal tegmentum nuclei and the ventromedial medullary reticular formation are known to be a possible source of nitrergic input to the TMN (Pose et al., 2005; Travers et al., 2005). Thus, it is possible that NO modulates TASK channels expressed in the MNs in the dl-TMN.

### Effects of 8-Br-cGMP on IRs in small and large MNs in the dl-TMN

Under the current-clamp condition, 100 μm 8-Br-cGMP hyperpolarized the membrane potential and reduced the number of spikes evoked by a depolarizing current pulse in a small MN (Fig. 6*Aa*,*Ab*). Then, *I–V* relationships were measured at the peak timing of sag potentials and just before the offset of current pulses (see Materials and Methods). An inwardly rectifying current (*I*_ir_) became apparent at potentials more negative than −100 mV as represented by an inwardly rectifying *I–V* relationship measured just before the offset of current pulses (Fig. 6*Ac*, blue crosses), in contrast to the almost linear *I–V* relationship measured at the peak timing of sag potentials (Fig. 6*Ac*, blue circles). However, the slope of the linear regression line for the *I–V* relationship measured at potentials ranging between the resting membrane potential and −100 mV was almost the same as between those measured at the peak timing of sag potentials and before the offset of current pulses (Fig. 6*Ac*). From this rectifying potential at approximately −100 mV, it is also suggested that the *I*_ir_ is mediated by a voltage-dependent K^+^ current, instead of an h current. Thus, neither *I*_ir_ nor currents other than voltage-independent leak K^+^ currents are involved in the IR that was measured at the peak of sag potentials evoked in MNs of dl-TMN in response to the hyperpolarizing current pulses applied at the resting membrane potential.

Accordingly, the *I–V* relationships that were measured at peak sag potentials were compared before and after the application of 8-Br-cGMP (Fig. 6*Ac*, blue circles, red circles). The slope of the linear regression line for the *I–V* relationship obtained after 8-Br-cGMP application became less steep compared with that obtained before 8-Br-cGMP application, and the two regression lines crossed at approximately −95 mV (the equilibrium potential for K^+^). This observation clearly indicates that 8-Br-cGMP decreased the IR through an enhancement of leak K^+^ currents without causing contamination by other conductances. In contrast, the inwardly rectifying component was not markedly affected by 8-Br-cGMP (Fig. 6*Ac*, blue crosses, red crosses), suggesting that at potentials more hyperpolarized than −100 mV, where *I*_ir_ was activated, the enhancement of leak K^+^ current by 8-Br-cGMP was largely masked by *I*_ir_.

In a large MN, 8-Br-cGMP slightly depolarized the membrane potential and increased the number of spikes (Fig. 6*Ba*,*Bb*). The slope of the linear regression line for the *I–V* relationship obtained after 8-Br-cGMP application became slightly steeper compared with that obtained before 8-Br-cGMP application, and the two regression lines crossed at approximately −95 mV, indicating a slight increase in the IR due to an inhibition of leak K^+^ currents (Fig. 6*Bc*). These results strongly suggest that 8-Br-cGMP changed membrane properties differentially between small and large MNs through differential modulation of leak K^+^ currents. The pooled data analysis in 14 MNs revealed that 8-Br-cGMP decreased the IRs in MNs that have larger IRs, while it either hardly changed or slightly increased those in MNs that have smaller IRs (Fig. 6*C*). Subsequently, the changes in IRs by 8-Br-cGMP were inversely correlated with the IRs of MNs, as revealed by the significant negative correlation (*p* < 0.001, *r* = −0.89, Pearson’s correlation) between the two parameters (Fig. 6*C*).

**Figure 8.**
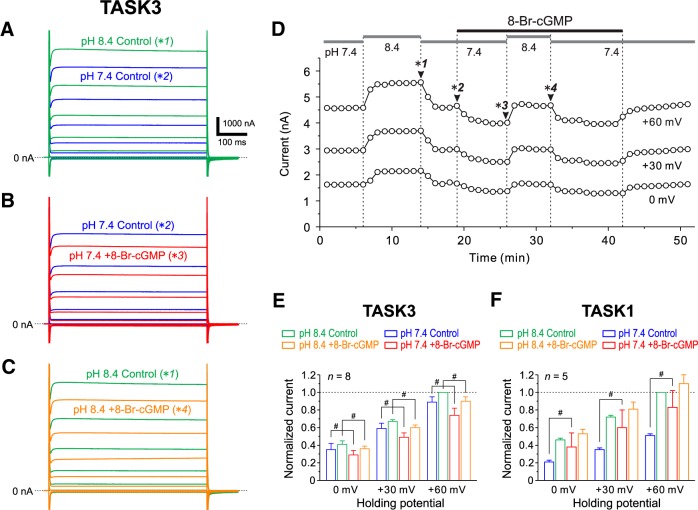
8-Br-cGMP inhibits cloned TASK3 channels. ***A***, Superimposed TASK3 currents evoked by voltage steps ranging between −150 and +60 mV in 30 mV steps applied at a holding potential of −90 mV at pH 8.4 and 7.4. The respective current traces were obtained at the time points indicated with *1 and *2 in ***D***. ***B***, Superimposed TASK3 currents obtained at pH 7.4 before (blue traces) and during (red traces) the application of 8-Br-cGMP. The respective current traces were obtained at the time points indicated with *2 and *3 in ***D***. ***C***, Superimposed TASK3 currents obtained at pH 8.4 before (green traces) and during the application of 8-Br-cGMP (orange traces). The respective current traces were obtained at the time points indicated with *1 and *4 in ***D***. ***D***, Plots of TASK3 currents at 0, +30, and +60 mV against time. Gray horizontal bars represent the timing and duration of perfusion of the extracellular solutions at respective pH, and the black horizontal bar represents the timing and duration of the addition of 100 μm 8-Br-cGMP. Arrowheads indicate the time points at which the respective current responses shown in ***A–C*** were obtained. ***E***, The normalized TASK3 currents recorded before and during the application of 8-Br-cGMP at pH 7.4 (blue and red columns, respectively) and those recorded before and during the application of 8-Br-cGMP at pH 8.4 (green and orange columns, respectively; *n* = 8). All of the TASK3 currents are normalized by that obtained before the application of 8-Br-cGMP at pH 8.4. #*p* < 0.05, two-way ANOVA followed by Fisher’s PLSD. ***F***, The normalized TASK1 currents recorded before and during the application of 8-Br-cGMP at pH 7.4 (blue and red columns, respectively) and those recorded before and during the application of 8-Br-cGMP at pH 8.4 (green and orange columns, respectively; *n* = 5). All of the TASK1 currents are normalized by that obtained before the application of 8-Br-cGMP at pH 8.4. #*p* < 0.05, two-way ANOVA followed by Fisher’s PLSD.

The effects of 8-Br-cGMP on IRs in large MNs were unexpected because TASK1 channels were more largely expressed in large MNs compared with small MNs (Fig. 1). However, if TASK3 channels expressed in the dendrites of large MNs are suppressed by 8-Br-cGMP, it is possible that 8-Br-cGMP does not necessarily change IRs in large MNs, presumably due to the opposing effects of 8-Br-cGMP on TASK1 and TASK3 channels. If this is the case, the IRs of dendritic membrane in large MNs would be increased through the suppression of TASK3 channels. To examine this possibility, we next investigated the effects of 8-Br-cGMP on EPSPs evoked by a single electrical stimulation applied just dorsal to or in the dorsal part of the TMN in large MNs. Indeed, the amplitudes of EPSPs evoked in larger MNs that have small IRs were increased after the application of 100 μm 8-Br-cGMP (Fig. 6*D*; also see Fig. 6*E*), suggesting that TASK3 channels expressed in dendrites of large MNs are suppressed by 8-Br-cGMP. In contrast, 8-Br-cGMP decreased the amplitudes of EPSPs in smaller MNs that have larger IRs (Fig. 6*E*). The pooled data analysis in 13 MNs revealed that the changes in EPSP amplitude by 8-Br-cGMP were significantly inversely correlated with the IRs of MNs (Fig. 6*E*; *p* = 0.002, *r* = −0.76, Pearson’s correlation). Taken together, these results suggest that 8-Br-cGMP decreases IRs in smaller MNs in the dl-TMN due to the activation of TASK1 channels, while it either hardly changes or slightly increases those in larger MNs in the dl-TMN, presumably due to the opposing effects of 8-Br-cGMP on TASK1 and TASK3 channels (Fig. 8; Discussion).

### 8-Br-cGMP modulates the latency to the first spike in MNs in the dl-TMN

We next examined the effects of 8-Br-cGMP on the rank-ordered recruitment induced by repetitive electrical stimulation of Ia afferents in a pair of MNs in the dl-TMN, which were simultaneously recorded. 8-Br-cGMP decreased the IR and hyperpolarized the resting membrane potential, which consequently increased the latency to the first spike in a smaller MN (Fig. 7*A*, compare green traces, red traces). In contrast, 8-Br-cGMP did not markedly change the IR or the resting membrane potential and slightly decreased the latency to the first spike in a larger MN (Fig. 7*B*). Consequently, this pair of MNs displayed almost the same latency to the first spike (Fig. 7*A*,*B*, compare arrowheads).

In the four pair recordings obtained from two MNs of different sizes that were visually identified under Nomarski optics, the IR and the latency to the first spike were measured before and after the application of 8-Br-cGMP and were plotted in the two-dimensional space defined by the IR and the latency to the first spike (Fig. 7*C*). The smaller MNs were invariably larger in the IR and shorter in the latency to the first spikes compared with the larger MNs in any pair recordings. The mean values of the IRs and the latencies to the first spikes were significantly (*p* = 0.045 and *p* = 0.029, respectively, paired *t* test) different between the smaller and larger MNs.

The discrimination analysis revealed that 8-Br-cGMP significantly (*p* = 0.032, Wilks’ lambda) changed the distribution of the IR and the latency to the first spike in the two-dimensional space in smaller MNs (Fig. 7*C*, blue open circles, red filled circles). The mean value of the latencies to the first spike was significantly (*p* = 0.045, paired *t* test) increased from 31.3 ± 2.9 to 39.5 ± 3.1 ms following the application of 8-Br-cGMP (Fig. 7*C*), although there was no significant (*p* = 0.129, paired *t* test) difference in the mean value of the IRs obtained before and after the application of 8-Br-cGMP (309 ± 75 and 290 ± 61 MΩ, respectively). In contrast, in larger MNs, 8-Br-cGMP did not significantly (*p* = 0.676, Wilks’ lambda) change the distribution of the IR and the latency to the first spike (Fig. 7*C*, blue open triangles, red filled triangles). There were no significant differences either in the mean value of the IRs (*p* = 0.101, paired *t* test) or in the mean value of the latencies to the first spikes (*p* = 0.140, paired *t* test) obtained before (230 ± 47 MΩ and 49.3 ± 5.1 ms, respectively) and after (243 ± 49 MΩ and 45.8 ± 6.9 ms, respectively) the application of 8-Br-cGMP.

Before the application of 8-Br-cGMP, the distribution of the IR and the latency to the first spike in smaller MNs were significantly (*p* = 0.006, Wilks’ lambda) different from those in larger MNs (Fig. 7*C*, blue open circle and triangle). However, following the application of 8-Br-cGMP, there was no significant (*p* = 0.298, Wilks’ lambda) difference in the distribution of the IR and the latency to the first spike between the smaller and larger MNs. Subsequently, the mean value of the difference in the latency to the first spike between the smaller and larger MNs was significantly (*p* = 0.029, paired *t* test) decreased from 18.0 ± 4.2 to 6.3 ± 8.9 ms (Fig. 7*C*, compare blue double-headed arrows, red double-headed arrows). These results suggest that 8-Br-cGMP decreases the time necessary for the recruitment starting with smaller MNs and progressing to larger MNs, causing a more synchronous activation of smaller and larger MNs in the dl-TMN.

### 8-Br-cGMP inhibits TASK3 currents

In small MNs that primarily express TASK1 channels (Figs. 1, 2), 8-Br-cGMP decreased the IRs (Fig. 6*A*,*C*). Because it has been previously shown that cloned TASK1 channels are activated by 8-Br-cGMP (Toyoda et al., 2010), it is strongly suggested that the decreases in IRs in small MNs are mediated by the activation of TASK1 channels. However, 100 μm 8-Br-cGMP did not necessarily decrease IRs in large MNs (Fig. 6*C*) despite the higher expression of TASK1 channels in large MNs compared with small MNs (Fig. 1*F*). Because large MNs possess many dendrites that express TASK3 channels (Fig. 2*E*) and because 8-Br-cGMP increased the amplitudes of EPSPs (Fig. 6*E*), it is possible that 8-Br-cGMP increases the IRs of dendritic membrane by inhibiting TASK3 channels. Therefore, we next investigated whether and how 8-Br-cGMP modulates cloned TASK3 channels that are heterologously expressed in *Xenopus* oocytes.

TASK3 currents were evoked by voltage steps ranging between −150 and +60 mV by 30 mV steps applied at a holding potential of −90 mV. When the extracellular pH was decreased from 8.4 to 7.4, TASK3 currents evoked in response to positive voltage steps ranging between 0 and +60 mV were markedly decreased (Fig. 8*A*,*D*). When 100 μm 8-Br-cGMP was bath applied, TASK3 currents in response to the positive voltage steps ranging between 0 and +60 mV at pH 7.4 and 8.4 were markedly decreased (Fig. 8*B*,*C*, respectively; also see Fig. 8*D*). The pooled data analysis revealed that TASK3 currents at 0, +30, and +60 mV were significantly decreased by 8-Br-cGMP at pH 7.4 (0 mV, *p* = 0.019; +30 mV, *p* < 0.001; +60 mV, *p* < 0.001, two-way ANOVA followed by Fisher’s PLSD) and at pH 8.4 (0 mV, *p* = 0.044; +30 mV, *p* = 0.001; +60 mV, *p* = 0.001, two-way ANOVA followed by Fisher’s PLSD; Fig. 8*E*, *n* = 8). As it has previously been reported (Toyoda et al., 2010) that 8-Br-cGMP enhances TASK1 currents heterologously expressed in HEK293 cells at pH 7.3, we confirmed that 8-Br-cGMP enhances TASK1 currents in *Xenopus* oocytes in which cloned TASK1 channels were heterologously expressed. TASK1 currents at 0, +30, and +60 mV were significantly increased by 8-Br-cGMP at pH 7.4 (0 mV, *p* = 0.007; +30 mV, *p* = 0.003; +60 mV, *p* < 0.001, two-way ANOVA followed by Fisher’s PLSD), but not at pH 8.4 (0 mV, *p* = 0.228; +30 mV, *p* = 0.214; +60 mV, *p* = 0.161, two-way ANOVA followed by Fisher’s PLSD; Fig. 8*F*, *n* = 5). Based on these results, it can be concluded that 8-Br-cGMP enhances TASK1 currents, whereas it suppresses TASK3 currents.

### Effects of 8-Br-cGMP on spatiotemporal patterns of excitation spreads in the dl-TMN

**Figure 9.**
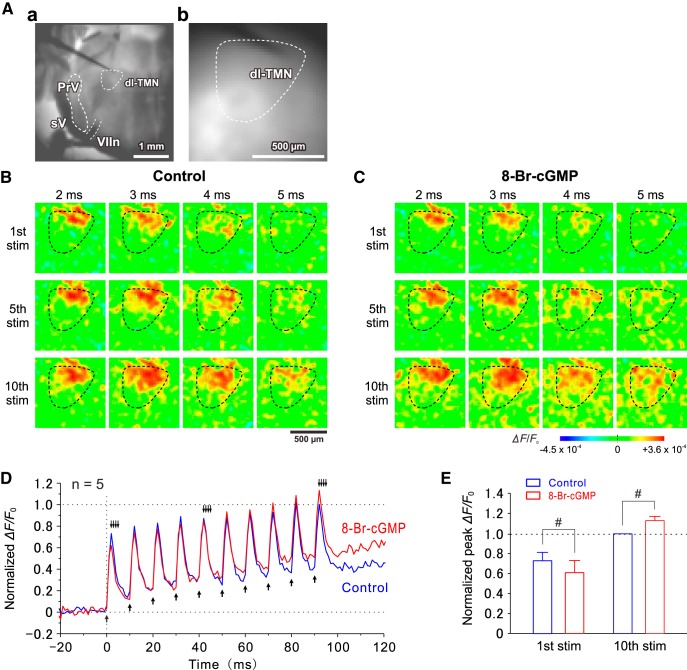
Recruitment of MNs in the dl-TMN caused by the stimulation of presumed Ia afferents. ***Aa***, A bright-field image of a brainstem slice including the dl-TMN. ***Ab***, A resting light intensity image of the dl-TMN. ***B***, ***C***, Sample pseudocolor images of optical responses induced by stimulation of the dorsal part of the TMN before (***B***) and after (***C***) the application of 8-Br-cGMP. ***D***, Traces representing averaged temporal profiles obtained before and after the application of 8-Br-cGMP (*n* = 5). Upward arrows indicate the time points at which the stimuli were applied. Downward arrows indicate the time points at which the respective pseudocolor images were obtained (***B***, ***C***). ***E***, The normalized peak amplitudes of optical responses induced by the 1st and 10th stimuli before and after the application of 8-Br-cGMP. #*p* < 0.02, two-way ANOVA followed by Fisher’s PLSD.

We next investigated by using voltage-sensitive dye imaging whether and how spatiotemporal patterns of excitation spreads that are evoked by repetitive electrical stimulations (10 pulses at 100 Hz) applied just dorsal to or in the dorsal part of the TMN (Fig. 9*A*) are modulated by 8-Br-cGMP. The excitation spread was gradually facilitated over the dl-TMN as the stimulation was repeated at 100 Hz (Fig. 9*B*). This frequency facilitation is characteristic of the Ia synapse on trigeminal MNs (Grimwood and Appenteng, 1995), in contrast to the well established frequency depression of Ia-EPSPs in spinal MNs (Curtis and Eccles, 1960). The temporal profile of the optical responses (Fig. 9*D*, control) revealed that the temporal summation of the optical responses occurred to recruit MNs in the dl-TMN. Following bath application of 100 μm 8-Br-cGMP, the excitation spread in response to the first stimulation appeared to be decreased compared with the control responses (Fig. 9*B*,*C*, first stimulation), whereas the excitation spread in response to the 10th simulation appeared to be enhanced compared with the control response (Fig. 9*B*,*C*, 10th stimulation). As revealed by the averaged temporal profiles obtained before and after the application of 8-Br-cGMP in the five slices examined (Fig. 9*D*), 8-Br-cGMP decreased the peak amplitudes of early optical responses but increased those of late optical responses to 10 successive stimulations. The peak amplitude of optical responses induced by the first stimulation after application of 8-Br-cGMP (0.61 ± 0.12) was significantly smaller (*p* = 0.015, two-way ANOVA followed by Fisher’s PLSD) compared with that obtained before the application of 8-Br-cGMP (0.73 ± 0.08), while that induced by the 10th stimulation after the application of 8-Br-cGMP (1.13 ± 0.04) was significantly larger (*p* = 0.007, two-way ANOVA followed by Fisher’s PLSD) compared with that obtained before the application of 8-Br-cGMP (1; Fig. 9*E*, *n* = 5). Thus, 8-Br-cGMP suppressed early optical responses but facilitated late optical responses, indicating the inhibition of the earlier recruitment of smaller MNs and the facilitation of later recruitment of larger MNs, respectively, causing a more synchronous activation of small and large MNs in the dl-TMN. This is consistent with the effects of 8-Br-cGMP on the IRs of smaller and larger MNs and on EPSPs (Fig. 6), and also with the observations made by simultaneous whole-cell recordings from a pair of small and large MNs (Fig. 7). Thus, TASK channels play crucial roles in the rank-ordered recruitment of MNs in the dl-TMN.

## Discussion

### TASK1/TASK3 mRNA expression levels and TASK1/TASK3 subunit distribution in small and large MNs in the dl-TMN

The immunohistochemistry revealed that TASK1 and TASK3 channels were complementarily distributed in soma and dendrites of MNs, respectively (Fig. 2), suggesting that there are few heterodimeric TASK1/3 channels in MNs in the dl-TMN. Then, the IR of MNs in the dl-TMN is likely to be determined primarily by the expression level of TASK1 channels. The real-time RT-PCR revealed that the expression level of TASK1 channels in one large MN was 2.1 times higher than that in one small MN (Fig. 1*F*), mediating lower and higher IRs in larger and smaller MNs, respectively. Because the mean diameters of dissected small and large MNs are 15–20 and >35 µm, respectively, the mean surface area in one large MN is approximately three times larger than that in one small MN. Then, the mean density of TASK1 channels in the soma of one small MN is estimated to be ∼1.4 times larger than that in one large MN, suggesting that the density of TASK1 channels increases with a decrease in cell diameter, in contrast to the general assumption that the density of leak K^+^ channels is invariable irrespective of cell sizes.

In the present study, TASK3 mRNA was hardly detected in small MNs, while TASK3 mRNA was detected half as much as TASK1 mRNA in one large MN (Fig. 1*F*). However, the expression level of TASK3 mRNA in large MNs may have been overestimated compared with that in small MNs because thinner dendrites expressing TASK3 in smaller MNs had been cut by LMD, whereas thicker proximal dendrites expressing TASK3 in larger MNs were well preserved as a part of the soma. Therefore, the difference in the relative value of TASK3 mRNA to TASK1 mRNA between small and large MNs may be smaller than the estimated value. In contrast with the present findings, previous studies (Karschin et al., 2001; Talley et al., 2001) using *in situ* hybridization demonstrated that the expression level of TASK3 mRNA in MNs appeared to be higher than that of TASK1 mRNA. This discrepancy can be explained by the underestimation of TASK3 mRNA levels in the present study because dendrites expressing TASK3 had been cut by LMD (Fig. 1). The group Ia synapses are made mostly on the dendrites of αMNs in the dl-TMN (Dessem and Taylor, 1989; Yabuta et al., 1996). As TASK3 channels were predominantly expressed in the dendrites of MNs (Fig. 2*E*), dendritic Ia-EPSPs would be modulated largely by the activity of TASK3 channels (Fig. 6*D*,*E*). Thus, our results suggest that IRs in small αMNs can be modulated largely by the activity of somatic TASK1 channels, while IRs in large αMNs can be modulated largely by the activity of TASK1 and TASK3 channels. The impact of synaptic inputs can be modulated by the activity of dendritic TASK3 channels in αMNs in the dl-TMN, which may be more prominent in large αMNs compared with small αMNs.

### The population of small MNs innervating JC muscles does not necessarily represent γMNs

It has been reported (Rokx et al., 1985, 1987) that soma diameters of rat MNs innervating JC muscles displayed a bimodal distribution showing two peaks at 15 and 24 μm, with the boundary at 20 μm, and the populations of small and large MNs were considered to be γMNs and αMNs, respectively. This is because the MNs included in the ventromedial TMN, which innervates jaw-opening muscles, displayed a unimodal distribution due to the absence of γMNs (Rokx et al., 1985, 1987). In the present study, we aimed to obtain whole-cell recordings from large and small MNs that have polygonal or stellate-shaped somata with many dendrites (Fig. 3), but not those showing piriform or round-shaped somata with fewer primary dendrites, which are the characteristics of γMNs (Card et al., 1986; Bae et al., 2002). Simultaneous recordings from small and large MNs revealed that Ia-like EPSPs were evoked in both small and large MNs simultaneously, and the small and large MNs were recruited in the size-dependent manner. These features of MNs are distinct from morphological and electrophysiological features of γMNs (Eccles et al., 1960; Moschovakis et al., 1991). Thus, both the small and large MNs in which whole-cell recordings were made are most likely to be αMNs.

On the other hand, we cannot rule out the possibility that in the real-time PCR study we sampled γMNs as small MNs (15–20 μm). The expression level of TASK1 channels in one large αMN is 2.1 times higher than that in one small αMN, provided that there was no difference in the expression level of TASK1 mRNA between γMNs and small αMNs (Fig. 1*F*). However, provided that the expression level of TASK1 channels is higher in γMNs than in small αMNs, the expression level of TASK1 mRNA in small αMNs may have been overestimated. On the contrary, provided that the expression level of TASK1 channels is lower in γMNs than in αMNs, the expression level of TASK1 mRNA in small αMNs may have been underestimated. Thus, the value of 2.1 times might be changed depending on the expression level of TASK1 mRNA in γMNs, although this value may not be <1, provided that there are at least as many αMNs as γMNs in the population of small MNs. Indeed, the population of small MNs was found to contain as many αMNs as γMNs, which were identified immunohistochemically using NeuN, Err3, and ChAT (Saito et al., 2013).

### Critical involvement of TASK channels in rank-ordered recruitment of αMNs in the dl-TMN

During the slow-closing phase of the mastication cycle (Lund, 1991), the isometric contraction of JC muscles is developed through the rank-ordered recruitment of αMNs innervating JC muscles (Yemm, 1977). In the present study, we demonstrated that the size of MNs is proportional to the expression level of somatic TASK1 channels (Fig. 1), and the IR is consequently larger in small αMNs than in large αMNs (Fig. 3). Furthermore, dual whole-cell recordings obtained from a pair of αMNs of different sizes revealed that the spike threshold in the smaller αMN is lower than that in the larger αMN (Fig. 4) and that αMNs are invariably recruited from the smaller to larger αMNs in response to repetitive stimulation of the presumed Ia afferents (Figs. 5, 7). These findings suggest that at least somatic TASK1 channels are critically involved in the rank-ordered recruitment of αMNs in the dl-TMN. This possibility was further confirmed by the modulation of TASK channels by 8-Br-cGMP.

In contrast with the enhancing effects of 8-Br-cGMP on cloned TASK1 currents (Toyoda et al., 2010), cloned TASK3 channels were found to be inhibited by 8-Br-cGMP (Fig. 8). Thus, TASK1 and TASK3 channels are inversely regulated by the cGMP–PKG signaling pathway. It has been reported that NO/cGMP signaling inhibited TASK-like currents in hypoglossal MNs (Wenker et al., 2012). Because it was demonstrated that 34% and 52% of leak K^+^ currents in hypoglossal MNs are mediated by the homodimeric TASK3 and heterodimeric TASK1/3 channels, respectively (Berg et al., 2004), the inhibitory effects of TASK-like currents by NO–cGMP–PKG pathway may have been brought about at least partly by the inhibition of homodimeric TASK3 channels, although it remains unknown how the NO–cGMP–PKG pathway modulates heterodimeric TASK1/3 channels. While it is well established that a rank-ordered recruitment of motor units occurs when increasing the bite force during voluntary isometric contraction of JC muscles (Yemm, 1977), to the best of our knowledge, there is no report showing that rank-ordered recruitment is involved in the control of tongue movement, probably because any isometric contraction does not take place in tongue muscles that have free ends. This functional difference may be reflected in the differential expression patterns of TASK channels between the αMNs in the dl-TMN and hypoglossal MNs. If this is the case, TASK1 channels may play an important role in regulating the isometric contraction through rank-ordered recruitment of αMNs compared with TASK3 or TASK1/3 channels.

Previous studies proposed that the specific membrane resistance, but not the cell size itself, is the key determinant of the orderly recruitment of spinal αMNs (Gustafsson and Pinter, 1984a,b). This hypothesis is strongly supported by the present findings that TASK channels are differentially expressed in the small and large MNs (Fig. 1), because the density of TASK channels directly impacts membrane resistance. The expression levels of TASK channels rather than cell size itself appear to play a more fundamental role in the orderly recruitment of MNs in the dl-TMN, although the expression level of TASK1 is proportional to the size in MNs of the dl-TMN.

The orderly recruitment of MNs is determined not only by the intrinsic properties of MNs (Gustafsson and Pinter, 1985) but also by the synaptic inputs they receive (Heckman and Binder, 1988, 1993). Indeed, it has been demonstrated that not only Ia-EPSPs but also effective Ia-EPSCs were larger in higher IR MNs than in lower IR MNs, and the Ia synaptic input was estimated to produce a twofold expansion of the differences in MN recruitment thresholds that are generated by intrinsic cellular properties (Heckman and Binder, 1988). This finding is consistent with previous studies showing that the number of Ia synaptic terminals was larger (Burke, 1968) and that their release probabilities were higher (Honig et al., 1983; Collins et al., 1984) in MNs with higher IR than in MNs with lower IR, although these differences were not large (Peshori et al., 1998). If this is the case, the larger effective Ia-EPSCs in higher IR MNs may serve to increase the safety factor to ensure the orderly recruitment of MNs even under the condition that TASK1 and TASK3 channels are fully activated and suppressed, respectively, by 8-Br-cGMP.

### 8-Br-cGMP modulates the rank-ordered recruitment of αMNs through differential modulation of small and large αMNs

In small αMNs, 8-Br-cGMP hyperpolarized the resting membrane potentials and decreased the IRs (Figs. 6*A*, 7*A*), and subsequently increased the latencies to the first spikes (Fig. 7*A*,*C*). As TASK1 channels are expressed in the somata of MNs (Figs. 1, 2), these results could be due to the enhancement of TASK1 currents by activation of the NO–cGMP–PKG pathway. In contrast, in large αMNs, 8-Br-cGMP hardly or slightly depolarized the resting membrane potentials and hardly or slightly increased the IRs (Figs. 6*B*, 7*B*), subsequently causing no increase or a slight decrease in the spike-onset latencies (Fig. 7*B*,*C*). Because TASK1 and TASK3 channels are inversely regulated by 8-Br-cGMP (Fig. 8), the decreases in IRs through the activation of somatic TASK1 channels by 8-Br-cGMP may be canceled by increases in IRs through the inhibition of dendritic TASK3 channels by 8-Br-cGMP. However, the modest effect of 8-Br-cGMP on TASK3 compared with TASK1 channels appeared to be contradictory to the negligible effect of 8-Br-cGMP on the IRs in large αMNs. Nevertheless, this was not the case. TASK3 currents recorded in *Xenopus* oocytes were 5 to 10 times larger compared with TASK1 currents despite the fact that the number of cRNAs for TASK1 injected into *Xenopus* oocytes was the same as that for TASK3 (see Materials and Methods). This suggests that the expression level of TASK3 channels might have been much higher than that of TASK1 channels in *Xenopus* oocytes. Provided that PKG activation by 8-Br-cGMP is not enough to phosphorylate all the TASK3 channel substrates in single *Xenopus* oocytes, the effects of 8-Br-cGMP on TASK3 currents would be small. Thus, it is not reasonable to directly compare the effects of 8-Br-cGMP on TASK1 currents with that on TASK3 currents in *Xenopus* oocytes. Therefore, the modest effect of 8-Br-cGMP on TASK3 channels compared with TASK1 channels is not necessarily contradictory to the negligible effect of 8-Br-cGMP on the IRs in large αMNs. These results suggest that 8-Br-cGMP decreases the time necessary for recruitment starting with smaller αMNs and progressing to larger αMNs (Fig. 7*C*). Consistently, the voltage-sensitive dye imaging revealed that 8-Br-cGMP suppressed early optical responses but facilitated late optical responses to 10 repetitive stimulations applied at 100 Hz, indicating inhibition of the recruitment of smaller αMNs and the facilitation of the recruitment of larger αMNs, respectively (Fig. 9).

**Figure 10.**
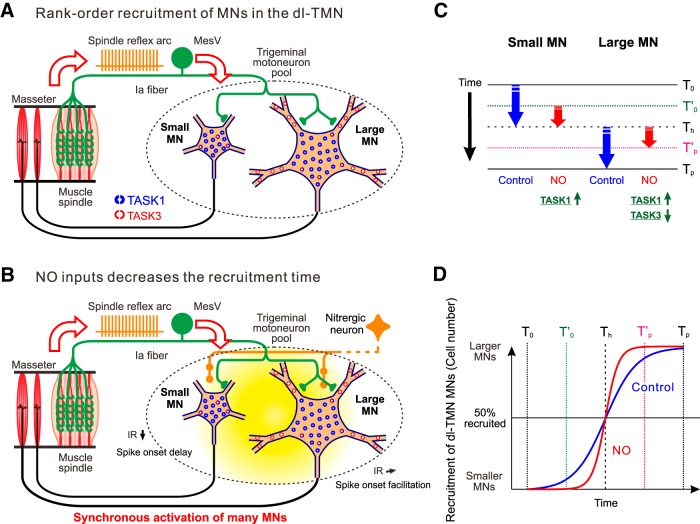
Rank-ordered recruitment of αMNs in the dl-TMN and its modulation by NO inputs. ***A***, During the isometric contraction of JC muscles, αMNs in the dl-TMN are recruited in rank order from smaller to larger αMNs in response to the activation of Ia afferents. ***B***, When NO is released in the TMN by the activity of nitrergic neurons, IRs in small αMNs are decreased, and subsequently increase the spike-onset latencies. By contrast, in relatively large αMNs, NO either hardly changes or slightly increases IRs, subsequently causing no increase or a slight decrease in the spike-onset latencies, while it facilitates recruitment of the largest αMNs. Consequently, NO causes a more synchronous activation of smaller and larger αMNs. ***C***, Alteration of the recruitment of small and large αMNs in the dl-TMN by the activation of NO inputs. T_0_, Recruitment-onset time; T_h_, time at which half of αMNs are recruited and from which large αMNs start to be recruited; T_p_, time at which all the αMNs are recruited; T’_0_, recruitment onset time after the activation of NO inputs; T’_p_, time at which all the αMNs are recruited after the activation of NO inputs. ***D***, Schematic diagram of the slow and fast rank-ordered recruitment observed before and after the activation of NO inputs, respectively. Slow precise and fast ballistic force increases can be achieved through the modulation of TASK1/TASK3 channels by NO inputs.

Together, given NO release in the TMN from nitrergic neurons during the isometric contraction of JC muscles (Fig. 10*A*), the spike-onset latency would increase in small αMNs, but would slightly decrease or remain unchanged in large αMNs, consequently decreasing the time necessary for the entire recruitment of αMNs to lead to a synchronous activation of many αMNs in the dl-TMN (Fig. 10*B–D*). To execute a ballistic movement or muscle contraction, many αMNs must be activated almost synchronously. Thus, slow and precise or fast and ballistic clenching may be achieved by the modulation of rank-ordered recruitment through the modulation of TASK1/TASK3 channels presumably by NO inputs. Based on the findings that 8-Br-cGMP almost exclusively affected the leak K^+^ currents without marked effects on the *I*_ir_ (Fig. 6*Ac*), it is strongly suggested that TASK channels play crucial roles in the rank-ordered recruitment of MNs in the dl-TMN. However, it should be noted that the linkage between TASK channels and the orderly recruitment of MNs derived from the findings obtained by using 8-Br-cGMP may be less direct compared with a demonstration using TASK channel knock-out mice.

## Synthesis

The decision was a result of the Reviewing Editor Douglas Bayliss and the peer reviewers coming together and discussing their recommendations until a consensus was reached. A fact-based synthesis statement explaining their decision and outlining what is needed to prepare a revision is listed below. The following reviewer(s) agreed to reveal their identity: Scott Chandler

Two reviewers have provided comments on this manuscript and both were quite positive about technical aspects of the work, which was performed to a high standard. However, two major issues with the manuscript will need to be addressed.

First, some historical references should be consulted for factors that influence recruitment order, both intrinsic and synaptic, and those earlier findings should be considered in discussing the implications of the present results. The references are noted in the first review.

Second, the data linking TASK channel expression with differences in various membrane properties is correlative - no matter how good the correlation, or how strong the rationale, the work falls short of an actual "demonstration" that TASK channels are responsible for the differences observed. Similar issues were raised in the original J Neuroscience reviews. In light of this, the conclusions that TASK channels account for the measured differences should be toned down throughout the manuscript, perhaps with changes to the title and introduction. Some of the shortcomings in this evidence should be acknowledged explicitly, so that the presentation is more circumspect in this regard. The second review provides some specific areas of concern and suggestions for revision.

Reviewer #1

Advances the Field

This is the first new work on mechanisms of orderly recruitment in many years. This recruitment process is fundamental to all motor output.

Comments to the Authors

This is a remarkable manuscript, a tour de force technically. The results have profound implications for our understanding of orderly recruitment in motor nuclei, which is the fundamental basis of all motor output. The authors clearly articulate on these implication, focusing naturally on their effective identification of a major role for TASK 1 and 3 channels. They miss other implication as they appear to have very limited knowledge of previous studies on the mechanisms of orderly recruitment that were carried out in the 1980's and 90's on spinal motoneurons (see below). This oversight is easily corrected however and overall this work constitutes an important and provocative contribution. I do not agree with previous reviewers that this work fails to demonstrate a clear link between TASK channels and recruitment - this link appears to me to be strongly supported by the results.

Major:

It is essential that the authors read the pair of papers by Gustafsson and Pinter, published in J. Physiol in 1984. This work argued that specific membrane resistance (Rm), not cell size, was the key determinant of recruitment order. The present papers results showing differential expression of TASK channels clearly supports this interpretation as TASK channels density directly impacts Rm. The correlation between Rm and IR (as the authors refer to input resistance) then results in a more than 10 fold range in rheobase (the injected current required to evoke a spike). In contrast to the present results, differences in spike voltage threshold were small. Thus the present results suggest either that the previous in vivo results were flawed and failed to detect spike voltage threshold differences or that trigeminal motoneurons are different than spinal motoneurons in this regard. In any case, it seems likely that measurements of rheobase currents were made in trigeminal neurons, the range would be especially wide as cell size and Rm determine IR; and IR in combination with differences in spike voltage threshold would require much larger currents in large than small motoneurons. The emphasis on rheobase current (or on threshold current for repetitive firing) is important, because this parameter can compared directly to synaptic currents (PSPs are less helpful, as their amplitudes are synaptic current x IR - or for transient EPSPs, input impedence).

The second major point is that recruitment order is not solely determined by the intrinsic properties of motoneurons but also by their inputs. This point was emphasized by Burke and studies of synaptic currents from various sources by M.D. Binder and colleagues published in the late 80's and early to mid 90's showed that excitation is distributed non-uniformly amongst large and small motoneurons. For the present manuscript, it is particularly important that this is true of Ia afferent input (see Heckman and Binder 1988, J Neurophysiol). An important additional consideration is whether is there any tonic synaptic input in these slices. Presumably not, but it does not appear that this was evaluated. The interaction between intrinsic and input has been systematically analyzed (Heckman and Binder 1993, J. Neurophysiology).

Reviewer #2

Advances the Field

Limited. The results of most of the data presented is not surprising. The main data the reviewers were looking for was the role of TASK channels in orderly recruitment of motoneuron in the trigeminal motor pool. Their data is interesting but very indirect and correlative on that point. However, a much less surprising set of observations was that there is an orderly recruitment of MNs and there is a relationship between cell size and electrical properties such as Rinp and threshold and resting potential. Manuscript also provides direct evidence that there is an orderly synaptic recruitment of MN, presumably from 1A afferent terminals(however, not certain that they are definitive 1A terminals).

Comments to the Authors

1. Manuscript describes TASK1 and TASK3 channel expression in two groups of neurons: large and small motoneuron within trigeminal motor nucleus using laser capture and real time qpcr in conjunction with immunohistochemistry. In a group of arbitrarily defined preselected (small (15-20um and large (<35um) MNs they found large MNs express more TASK1 compared to small MNs. Authors do not show data from a continuum of cell size and TASK expression levels, this would probably be very difficult, but maybe choosing 2 additional intermediate cell sizes would be more informative about direct relationship between TASK expression levels and cell size.

2. Manuscript describes relationship between cell size and various electrical parameters such as Rinp, resting potential and spike threshold using patch clamp methods. In particular they found the typical relationships expected, ie. Small cells have large Rinp, low threshold, compared to large cells. However, the relationship between the electrical properties and TASK expression is indirect and correlative.

3. Manuscript shows that small motoneurons are synaptically recruited before large ones (Henneman size principle) and have lower spike threshold. This observation of course is not surprising.

4. Manuscript describes modulation of TASK channels in oocytes by 8-Br-cGMP.

5. Manuscript describes the modulation of rank order recruitment by exogenous application of 8-Br-cGMP and its differential effects on small and large motoneurons. There is an implication that nitrous oxide could modulate TASK via cGMP since NO terminals are found on trigeminal motoneurons. However, isn't it reasonable to suggest that cGMP activation can have downstream effects on a variety ion channels in addition to TASK?

From the above separate studies the authors conclude that "TASK channels play a critical roles in of rank order recruitment of motoneuron within the dorsolateral trigeminal motor nucleus".

Each of the above experiments is very interesting and performed rigorously. However, the final conclusion that TASK channels are the critical determinants for orderly recruitment of MNs is correlative and indirect. Even if this were a demonstration, frankly I don't believe that conceptually it is very surprising. TASK channels have been demonstrated in motor pools previously as determinants of leak conductance, and thus, have been considered strong contributors to Rinp. Although conduction velocity is a typical proxy for cell size, in the 70s and 80s Burke's lab, D. Kernell's lab and probably others demonstrated the relationship between cell size and CV. Furthermore, Kernell also described the unexpected large conductance in large MN was probably a result of a decrease in specific membrane resistance as well (D. Kernell 1981, Brain research 204.).

It is curious that the title of the manuscript and the introduction stress role of TASK channels in orderly recruitment, yet the final concluding paragraph in the discussion section makes no mention of TASK channels but stresses 1) motoneurons are orderly recruited by Ia afferents (yes-demonstrated in this manuscript), and 2) the potential role of NO (not directly tested but strong implication), and 3) fast and slow ballistic clenching movements may be achieved by modulation of rank order recruitment by NO-cGMP-PKG pathway (Speculative but interesting possibility).

This is a nice series of individual experiments that are very interesting but I believe together they are not a convincing demonstration of the role of TASK expression in orderly recruitment of motoneurons.
